# Capsaicin mitigates ventilator-induced lung injury by suppressing ferroptosis and maintaining mitochondrial redox homeostasis through SIRT3-dependent mechanisms

**DOI:** 10.1186/s10020-024-00910-y

**Published:** 2024-09-12

**Authors:** Jinyuan Lin, Huajin Ou, Bijun Luo, Maoyao Ling, Fei Lin, Liming Cen, Zhaokun Hu, Liu Ye, Linghui Pan

**Affiliations:** 1 Department of Anesthesiology, Guangxi Medical University Cancer Hospital, He Di Rd No.71, Nanning, 530021 People’s Republic of China; 2 Guangxi Engineering Research Center for Tissue & Organ Injury and Repair Medicine, Nanning, China; 3 Guangxi Health Commission Key Laboratory of Basic Science and Prevention of Perioperative Organ Disfunction, Nanning, China; 4 Guangxi Clinical Research Center for Anesthesiology, Nanning, China

**Keywords:** Ventilation-induced lung injury, Capsaicin, Ferroptosis, Mitochondrial redox

## Abstract

**Background:**

Ventilator-induced lung injury (VILI) is one of the severe complications in the clinic concerning mechanical ventilation (MV). Capsaicin (CAP) has anti-inflammatory and inhibitory effects on oxidative stress, which is a significant element causing cellular ferroptosis. Nevertheless, the specific role and potential mechanistic pathways through which CAP modulates ferroptosis in VILI remain elusive.

**Methods:**

VILI was established in vivo, and the pulmonary epithelial cell injury model induced by circulation stretching (CS) was established in vitro. Both mice and cells were pretreated with CAP. Transmission electron microscopy, ELISA, Western blot, immunofluorescence, RT-PCR, fluorescent probes, and other experimental methods were used to clarify the relationship between iron death and VILI in alveolar epithelial cells, and whether capsaicin alleviates VILI by inhibiting iron death and its specific mechanism.

**Results:**

Ferroptosis was involved in VILI by utilizing in vivo models. CAP inhibited ferroptosis and alleviated VILI's lung damage and inflammation, and this protective effect of CAP was dependent on maintaining mitochondrial redox system through SITR3 signaling. In the CS-caused lung epithelial cell injury models, CAP reduced pathological CS-caused ferroptosis and cell injury. Knockdown SIRT3 reversed the role of CAP on the maintaining mitochondria dysfunction under pathological CS and eliminated its subsequent advantageous impacts for ferroptosis against overstretching cells.

**Conclusion:**

The outcomes showed that CAP alleviated ferroptosis in VILI via improving the activity of SITR3 to suppressing mitochondrial oxidative damage and maintaining mitochondrial redox homeostasis, illustrating its possibility as a novel therapeutic goal for VILI.

**Graphical Abstract:**

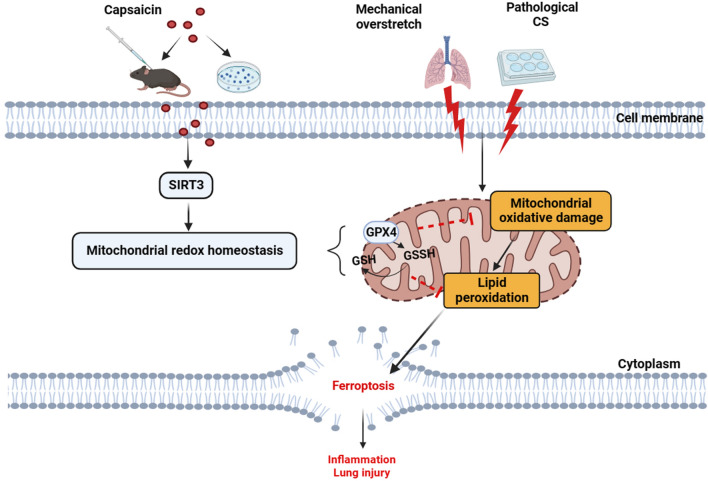

**Supplementary Information:**

The online version contains supplementary material available at 10.1186/s10020-024-00910-y.

## Background

High morbidity and mortality rates continue to pose significant challenges in acute respiratory distress syndrome (ARDS) (Bellani et al. 2016; Mart and Ware 2020). Mechanical ventilation (MV) serves as the primary management strategy for severe respiratory failure in ARDS (Tonetti et al. 2017; Paudel et al. 2021). Whereas inappropriate MV could either initiate or aggravate lung injury. This iatrogenic secondary lung injury due to MV is widely recognized to be ventilator-induced lung injury (VILI) (Slutsky and Ranieri 2013). VILI can amplify the systemic inflammatory response, potentially leading to multiple organ failure and even death, thus emerging as one of the most severe complications encountered during MV treatment (Fan et al. 2018). As such, there is an urgent need for a comprehensive understanding of VILI's mechanisms and innovative treatments.

Ferroptosis is current determined regulated form of necrosis, which is different from apoptosis, autophagy, necrosis, is instigated by plasma membrane damage, iron toxicity, lipid peroxidation (Tang et al. 2019; Chen et al. 2021a). The extreme buildup of reactive oxygen species (ROS) is termed oxidative stress (Wu et al. 2013). Excessive ROS production can reduce the reflection of glutathione peroxidases 4 (GPX4), thereby amplifying lipid oxidative harm, which are key elements precipitating ferroptosis (Dixon et al. 2012). The previous article indicates that MV with high tidal volume (HTV) can promote ROS production, thereby contributing to VILI in rats (Tang et al. 2021). Furthermore, we established a direct link between ferroptosis and VILI, showing that blocking ferroptosis in lung epithelial cells significantly reduced MV-associated lung injury in mice (Ling et al. 2023). These results emphasize the integral role of MV with HTV in promoting ferroptosis, which then leads to VILI; the detailed underlying mechanism, however, still need to be clarified.

The Sirtuin 3 (SIRT3) protein, one part of the Sirtuin protein group, is a class III deacetylase with NAD-dependent deacetylase activity (Brown et al. 2013). SIRT3 plays a crucial function in adjust of mitochondrial characteristic, notably by scavenging ROS and inhibiting lipoxygenase activity (Liu et al. 2017; Miller 2020). Additionally, our prior work showed that reduced SIRT3 levels led to heightened mitochondrial fission and oxidative damage, exacerbating lung ischemia–reperfusion injury in mice (Liu et al. 2022a). More and more papers have emphasized the important role of SIRT3 in the resistance to ferroptosis (Jin et al. 2021; Li et al. 2022).

Capsaicin (CAP), was called 8-methyl-N-geranyl-6-nonamide chemically, serves as the principal constituent of chili pepper (Srinivasan 2016). Numerous studies have documented the diverse biological properties of CAP, including analgesic effects (Derry et al. 2017), anti cardiovascular disease activity (He et al. 2017), anti-arthritis potential (Fattori et al. 2016), antiobesity properties (Zheng et al. 2017), and anticancer potential (Chapa-Oliver and Mejia-Teniente 2016). Among these, its anti-inflammatory effects have garnered significant attention. CAP not only mitigates inflammation in lipopolysaccharide-induced acute lung injury (ALI) (Chen et al. 2021b) but also moderates the inflammatory response in sepsis by targeting the PKM2-LDHA and COX-2 pathways (Zhang et al. 2022a). Moreover, CAP showcases protective effects against lipid peroxidation (Zhang et al. 2017) and oxidative stress-induced damage (Yashiro et al. 2015), both central to inhibiting ferroptosis. Recent findings indicate that CAP pre-treatment can deter oxidative stress and inflammation by preserving mitochondrial function, thereby protecting heart muscle cells from LPS-induced damage (Qiao, et al. 2021). Yet, the potential of CAP to counteract ferroptosis in VILI and its precise mechanism remain under-explored. Studies have verified that capsaicin could maintain the morphology and function of BAT against the whitening stimuli by activating the AMPK-SIRT3 to epigenetically inhibit MCU-dependent mitochondrial Ca^2+^ overload in brown adipocytes (Gao et al. 2020). However, there remains a lack of studies to reveal whether SIRT3 is involved in the potential of CAP to counteract ferroptosis in VILI.

In this study, our objective is to examine the protective properties of CAP on VILI both in vivo and in vitro and to delve deeper into the underlying molecular mechanisms. Collectively, our discoveries could spotlight a promising therapeutic agent adept at mitigating VILI.

## Methods

### Reagents

CAP (HY-10448), SIRT3 selective inhibitor 3-TYP (3-(1H-1, 2, 3-triazol-4-yl) pyridine, HY-108331) and Phen Green^™^ SK (PGSK,) diacetate were procured from MedChemExpress Company (Shanghai, China). The ferroptosis selective inhibitor ferrostain-1 (Fer-1) was sourced from Targetmol (Shanghai, China). Enzyme-linked immunosorbent assay (ELISA) kits for quantifying tumor necrosis factor-alpha (TNF-α), interleukin-6 (IL-6), and interleukin-1 beta (IL-1β) were obtained from Multisciences (Lianke) Biotech Company (Hangzhou, China). The tissue iron content assay kit and the mitochondrial membrane potential (MMP) fluorescent probe (JC-10) were acquired from Solarbio (Beijing, China). Kits for detecting total protein, malondialdehyde (MDA), glutathione (GSH), manganese superoxide dismutase (Mn-SOD), ATP, and ROS levels were purchased from Beyotime Biotechnology (Shanghai, China). C11- BODIPY^581/591^ and the cell counting kit-8 (CCK8) were procured from Glpbio (Montclair, CA, USA). SLC7A11 (12509; Affinity), GPX4 (sc-166570; Santa), SIRT3 (10099; Proteintech), and β-actin (4970; Cell Signaling Technology) were employed as primary antibodies. Goat anti-rabbit IgG H&L (ab216773) was sourced from Abcam (Cambridge, MA, USA), and the goat anti-mouse IgG H&L (92632210) from LI-COR (NE, USA). Cy3-labeled goat anti-rabbit IgG (GB21303) and Cy3-labeled goat anti-mouse IgG (GB21301) were purchased from Servicebio Technology Co., Ltd (Wuhan, China). Specific primers for SLC7A11, GPX4, and SIRT3 were synthesized and provided by Sangon Biotech (Shanghai, China).

### Establishment of the VILI mouse model

Male C57BL/6 J (wild-type) mice, aged 6–8 weeks and weighing approximately 25 ± 2 g, were procured from the Animal Center of Guangxi Medical University (Nanning, China). They were placed in controlled situations at indoor temperature of 22 °C ± 2 °C, a dark/light period of twelve hours, and free obtain standard water, food. Animals were randomly assigned to five groups: the control group (CON group, spontaneous breathing after tracheal intubation for 4 h), HTV group (HTV group, MV with HTV of 20 ml/kg for 4 h), HTV + Fer-1 group (Fer-1 [1 mg/kg] administered intraperitoneally for 14 consecutive days before MV [20 ml/kg for 4 h]) (Ling et al. 2023), CAP + HTV group (CAP administered intraperitoneally for 3 consecutive days at various concentrations [0.5, 1, or 2 ml/kg] before MV [20 ml/kg for 4 h]) (Chen et al. 2021b), and 3-TYP + CAP + HTV group (3-TYP [50 ml/kg] administered intraperitoneally every 2 days for a total of three times (Liu et al. 2022a; Zhai, et al. 2017), with CAP [1 ml/kg] administered on the final 3 days preceding MV [20 ml/kg for 4 h]). The VILI animal mode was developed according to our prior papers (Ye et al. 2020; Liao et al. 2021).

After concluding MV or spontaneous breathing, mice were euthanized through the intraperitoneal administration of a lethal anesthetic dose. Bronchoalveolar lavage fluid (BALF), Lung tissue, Blood serum, were then harvested for subsequent experimental analysis. It is crucial to highlight that all animal procedures must be executed with the utmost care to minimize inflammation induction.

### Histopathological analysis

The lung tissue was put in a 4% formaldehyde solution before being put in paraffin. These tissue parts were assigned to 4-µm-thick pieces and mounted onto slides. Since dewaxing, these slices were carefully stained with H&E, next, scrutinized under a light microscope. The extent of lung damage was assessed using standardized ALI protocols, which was demonstrated before (Matute-Bello et al. 2011).

### Immunofluorescence staining

Immunofluorescence was conducted as described previously (Zhang et al. 2017). Put simply, the lung tissue Sects. (4 µm) underwent immunofluorescent staining following a series of dewaxing and hydration. Primary antibodies against SLC7A11 (1:200 dilution), GPX4 (1:200 dilution), and SIRT3 (1:200 dilution) were applied. Cy3-labeled goat anti-rabbit IgG (1:300 dilution) or Cy3-labeled goat anti-mouse IgG (1:300 dilution) served as fluorophore-conjugated secondary antibodies. Nuclei were stained using DAPI. The stained lung tissue specimens were subsequently observed under a fluorescence microscope (Nikon EclipseC1, Nikon). The mean fluorescence intensity was analyzed with ImageJ 1.53.

### Inflammatory responses

A crude method to gauge lung exudation during VILI involves computing the wet/dry (W/D) ratio. The wet lung, specifically the middle lobe of the right lung, next, dried in a 60 °C oven for 48 h. Pulmonary permeability was assessed by quantifying total protein levels in the BALF supernatant using a bicinchoninic acid (BCA) assay. Cells in BALF were enumerated using a hemocytometer to gauge inflammatory infiltration. Concentrations of IL-1β, IL-6, and TNF-α in lung tissue were identified with ELISA kits, adhering to the producer's guidelines.

### Assessment of the *iron* level in vivo

To ascertain ferroptosis levels, the iron content in the lung tissue was examined using an iron assay kit according to the manufacturer’s recommendations.

### *Establishment of an *in vitro* model of VILI*

Mouse lung epithelial cells (MLE12) were propagated in Dulbecco’s modified Eagle’s medium supplemented with 10% fetal bovine serum and 1% penicillin/streptomycin (Invitrogen, USA), and maintained at a temperature of 37 °C with 5% CO_2_ in a humidified air environment. For a stable knockdown of the SIRT3 gene in MLE12 cells, lentiviral vectors GV248 and GV358 offered by Shanghai Zhong Qiao Xin Zhou Biotechnology Co., Ltd. (Shanghai, China) were employed. Following this, an in vitro VILI model was developed based on previously reported studies (Ling et al. 2023; Wang et al. 2011; Jing, et al. 2020; Carta et al. 2011). Briefly, cells of logarithmic growth phase were inoculated in standard density onto six-well BioFlex plate (Flexcell International). After 24 h of cultivation, cells underwent cyclic stretching (CS) using the FX 4000 T Flexercell Tension Plus system, which is equipped with a 25-mm loading station (Flexcell International, McKeesport, PA, USA), the frequency is 30 cycles/min (0.5 Hz), maintaining a 1:1 stretch/relaxation rate and a sine wave pattern. Pathological CS in our study was set at a 20% alteration in the basement membrane surface area, equating to 80% of the total lung capacity. Cells were stretched for 4 h at 37 °C in a 5% CO_2_ humidified incubator. The entire operation was rigorously managed using computer software.

To investigate whether CAP could curtail ferroptosis in cells experiencing pathological CS, cells in the 20% group were pre-treated with various concentrations of CAP (1, 10, and 100 μM) for 1 h (Zhou et al. 2020). After the cells were stretched for 4 h, cell pellets were harvested for next tests.

### Cell viability measurement

Cell viability was ascertained with the CCK-8 assay kit. Post respective treatments, cells were settled in 96-well plates for 12 h. The culture medium was replaced with fresh medium supplemented with 10% CCK-8 reagent and incubated at 37 °C for 1 h. The absorbance (optical density) was recorded with a microplate reader at 450 nm.

### *Evaluation of lipid oxidation *in vitro

Lipid oxidation in MLE12 cells was evaluated using C11-BODIPY^581/591^. Cells were treated with C11-BODIPY^581/591^ (20 μM) at 37 ℃ for 10 min and then washed with phosphate-buffered saline (PBS). Nuclei were stained with DAPI. Similarly, intracellular chelating iron was quantified using PGSK diacetate. Cells were treated with 20 μM PGSK at 37 °C for 15 min, and outcomes observed using fluorescence microscopy (Leica, Germany) and analyzed with ImageJ 1.53.

### Transmission *electron* microscopy

The procedures utilized were consistent with those described in in our past paper (Wu et al. 2013). Briefly, the lung tissue was procured within 1–3 min after modeling and then sectioned into 1–2 mm cubes. In 0.1 M sodium acetate buffer (pH 7.4), fixed at 4 ℃ for at least 2 h with 2.5% glutaraldehyde and 2.5% paraformaldehyde. The cubes were subsequently treated with the same buffer containing 1% osmium tetroxide for 2 h at room temperature, followed by dehydration in graded alcohols and propylene oxide. The specimens were put in resin, cut to ultrathin sections, stained with uranyl acetate and lead citrate, and identified under an HT7800 transmission electron microscope (Hitachi, Japan). Likewise, MLE12 cells were gathered for transmission electron microscopy (TEM) to inspect cell damage and mitochondrial ultrastructure.

### Single-cell suspensions

For ROS measurements, lung tissue was processed to prepare a monoplast suspension (Koppula et al. 2018). The whitening regions in the lungs were ground and subjected to digestion with 5000 U/mL collagenase type IV and 20 U/mL DNase at 37 °C for 40 min on a shaking bed. The resultant mixture was then filtered through a 100-mm cell strainer to secure single-cell suspensions.

### Evaluation of mitochondrial damage

ROS assay kit was utilized to detect ROS extents. Cells were incubated with 10 mM DCFH-DA at 37 °C for 30 min. After two washes with PBS, the fluorescence was quantified via flow cytometry. MMP was gauged using JC-10 staining, adhering to the producer's structure, and visualized utilizing a fluorescence microscope. Moreover, ATP concentrations were ascertained through firefly luciferase-associated chemiluminescence, in compliance with the producers' guidelines.

### Measurement of oxidative damage markers in mitochondria

Relative concentrations of MDA, Mn-SOD, and GSH in tissue or cell lysates were evaluated using the appropriate commercial kits. Notably, all experimental procedures were rigorously followed as per the manufacturer’s guidelines.

### Real-time polymerase chain reaction

mRNA expression levels for SLC7A11, GPX4, and SIRT3 were detected using real-time polymerase chain reaction (PCR) and specific primers on a real-time PCR system, as previously detailed (Wu et al. 2013). The primer sequences were as follows (5′ to 3′):

Mouse SLC7A11-F GCTGACACTCGTGCTATT.

Mouse SLC7A11-R ATTCTGGAGGTCTTTGGT.

Mouse Gpx4-F GCCTGGATAAGTACAGGGGTT.

Mouse Gpx4-R CATGCAGATCGACTAGCTGAG.

Mouse SIRT3-F CCACGACAAGGAGCTGCTTCTG.

Mouse SIRT3-R ACCCTGTCCGCCATCACATCA.

Mouse β-actin-F CCACGACAAGGAGCTGCTTCTG.

Mouse β-actin-R ACCCTGTCCGCCATCACATCA.

β-Actin was used as an internal standard for normalization, applying the 2^−△△Ct^ quantification method.

### Western blot analysis

Total proteins were isolated from lung tissue and MLE12 cells utilizing radio-immunoprecipitation assay buffer enriched with protease inhibitors. Concentrations were identified with the BCA protein assay kit. Equal amount of protein, along with the molecular weight marker, were loaded onto sodium dodecyl sulfate–polyacrylamide gels, next shifted to polyvinylidene fluoride film. Since blocking with a western blot rapid blocking buffer for 15 min at indoor temperature, the membranes were treated with primary antibodies for SLC7A11 (1:700 dilution), GPX4 (1:100 dilution), SIRT3 (1:1000 dilution), β-actin (1:1000 dilution). This was succeeded by incubation with secondary antibodies: either goat anti-rabbit IgG H&L (1:15,000 dilution) or goat anti-mouse IgG H&L (1:20,000 dilution). Finally, western blotting strips were visualized utilizing Odyssey two-color infrared laser imaging system (LICOR, America).

### Statistical analyses

SPSS 26.0 software (IBM, USA) was utilized for statistical analysis. All data were presented as mean ± standard deviation. Differences were assessed using analysis of variance, with subsequent least significant difference-t test and the Student–Newman–Keuls test for pairwise comparisons. A p-value of below 0.05 was deemed statistically significant.

## Results

### Ferroptosis was present during VILI

To identify ferroptosis was involved in VILI, we administered the ferroptosis inhibitor Fer-1 to the HTV group. As expected, MV with HTV increased the total iron (Supplementary Fig. 1A) and decresed the mRNA and protein degrees of SLC7A11 and GPX4 in the lung tissue (Supplementary Fig. 1B-E). From the TEM observations, we noted that the mitochondria in HTV group became smaller, the mitochondrial ridge decreased, and the mitochondrial membrane density increased which are considered characteristic morphological features of ferroptosis (Supplementary Fig. 1F). However, these effects were mitigated by Fer-1, as illustrated in the Fer-1 + HTV group (Supplementary Figure A-F). The information shows that ferroptosis plays a role in VILI. Furthermore, Fer-1 pretreatment significantly reduced lung edema and the degree of pathological injury, as compared with the HTV group. This was determined by evaluating the lung W/D ratios (Supplementary Fig. 1G), cell counts in BALF (Supplementary Fig. 1H), H&E staining, and lung injury scores (Supplementary Fig. 1I, J). Additionally, the levels of cytokine profiles in plasma, involving IL-1β, IL-6, and TNF-α, were dramatically lower in the Fer-1 + HTV group compared to the HTV group (Supplementary Fig. 1 K-M). These findings suggest that ferroptosis existed and exacerbated the inflammatory damage caused by MV with HTV.

### Capsaicin has a protective effect on lung injury and inflammation after HTV

**Fig. 1:**
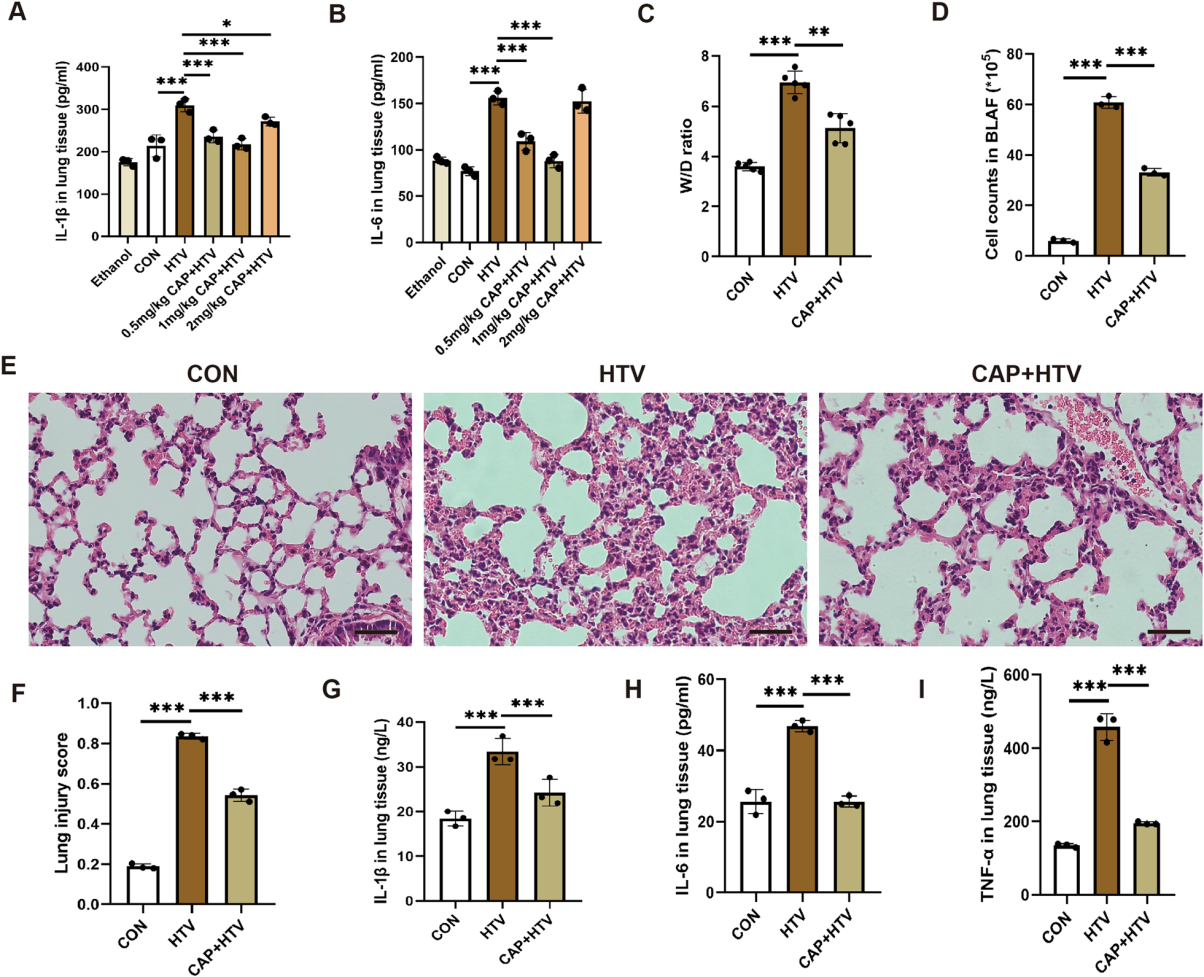
Capsaicin protected against lung injury and inflammation after HTV. **A** Levels of interleukin (IL)-1β in the lung tissue (n = 3). **B** Levels of IL-6 in the lung tissue (n = 3). **C** Wet/dry ratios of the lung tissue (n = 5). **D** Infiltrating cell counts in bronchoalveolar lavage fluid (n = 3). **E** Hematoxylin–eosin (H&E) staining in each group. Scale bar: 100 μm. **F** Pathological scores were assessed by H&E staining (n = 3). **G** Levels of IL-1β in the lung tissue (n = 3). **H** Levels of IL-6 in the lung tissue (n = 3). **I** Levels of tumor necrosis factor (TNF)-α in lung tissue (n = 3). Data are expressed as mean ± standard deviation. “*” indicates a significant difference between the corresponding groups (*p < 0.05, **p < 0.01 or ***p < 0.001)

To determine the optimal anti-inflammatory dose of CAP, ELISA kits were employed to evaluate serum levels of cytokine profiles, involving IL-1β (Fig. 1A) and IL-6 (Fig. 1B). As mentioned earlier, CAP was administered intraperitoneally for 3 consecutive days at various concentrations (0.5, 1, or 2 ml/kg) before MV. We determined that a concentration of 1 ml/kg of CAP was the most effective for anti-inflammation. Thus, this concentration was used in subsequent experiments. The results revealed that compared with the HTV group, the lung W/D ratios (Fig. 1C), cell counts in BALF (Fig. 1D), H&E staining, lung injury scores (Fig. 1E, F), and serum levels of cytokine profiles (Fig. 1G, H, I) in the CAP + HTV group were significantly decreased. These data suggest that CAP significantly alleviated lung injury and inflammation after HTV.

### Capsaicin further attenuated HTV-induced ferroptosis and mitochondrial oxidative damage in VILI

**Fig. 2:**
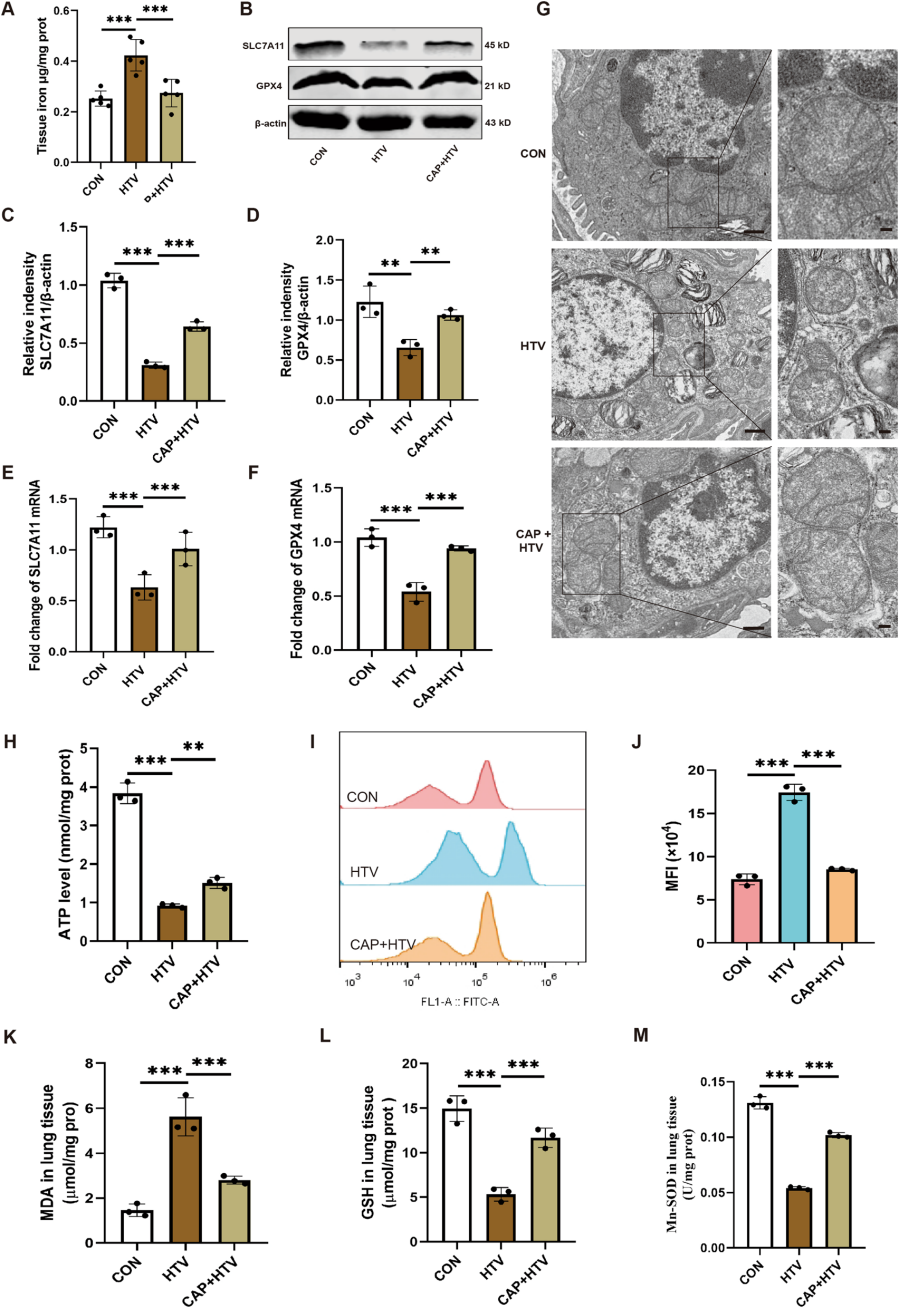
Capsaicin further attenuated HTV-induced ferroptosis and mitochondrial oxidative damage in VILI. **A** Iron levels in the lung tissue of the CON, HTV, and CAP + HTV groups (n = 5). **B** Representative western blots of SLC7A11, GPX4 and β-actin in the lung tissue (n = 3). **C**, **D** Relative protein expression of SLC7A11 and GPX4 was presented to β-actin. **E**, **F** Relative mRNA levels of SLC7A11 and GPX4 (n = 3). **G** Representative transmission electron microscopy images of lung tissue sections. Magnifications: 2000 × and acceleration voltage: 80 kV. Scale bar: 5.0 μm. Amplified pictures of mitochondria are labeled using black boxes, and the indicated area is shown at 6000 × magnification. Scale bar: 1.0 μm. **H** Amount of ATP in the lung tissue (n = 3). **I**, **J** Flow cytometry analysis of reactive oxygen species in monoplast suspension (n = 3). **K**–**M** Levels of malondialdehyde, glutathione, and Mn-SOD in the lung tissue (n = 3). Data are expressed as mean ± standard deviation. “*” indicates a significant difference between the corresponding groups (*p < 0.05, **p < 0.01, or ***p < 0.001)

To identify the influence of CAP on HTV-induced ferroptosis in vitro, we conducted several tests. Which displayed in Fig. 2, compared with the HTV group, iron overload, as indicated by rising iron levels (Fig. 2A) and lipid peroxidation, as indicated by an increase in MDA level (Fig. 2K) in the lung tissue decreased with CAP pretreatment under HTV conditions. Concurrently, the protein and mRNA levels of SLC7A11 and GPX4 were upregulated by CAP during HTV (Fig. 2B–F). Furthermore, TEM observations (Fig. 2G) revealed that mitochondria in lung tissue from the HTV group exhibited the morphological features characteristic of ferroptosis. However, this phenomenon was reversed in HTV mice pretreated with CAP. Collectively, these findings suggest that CAP ameliorated the ferroptosis induced by HTV.

What’s more, we assessed the impacts of CAP on mitochondrial oxidative damage in VILI by determining changes in ATP, ROS, MDA, GSH, and Mn-SOD levels. As expected, compared to the CON group, ATP degree was reduced, and ROS degree was significantly enhanced in HTV-treated mice (Fig. 2H–J). MDA levels, used to assess lipid peroxidation, was enhanced in the HTV-treated mice compared to the CON group (Fig. 2K). The mitochondrial antioxidant system was evaluated using GSH and Mn-SOD measurements. Notably, compared with CON group, the degrees of GSH and Mn-SOD in HTV group were dramatically reduced (Fig. 2L, M). As anticipated, CAP pretreatment prevented these effects, suggesting that CAP alleviated HTV-induced mitochondrial oxidative damage (Fig. 2). In summary, these findings suggested that CAP further mitigated mitochondrial oxidative damage in VILI.

### 3.4 Pharmacologic inhibition of SIRT3 alleviated the effect of capsaicin treatment on mitochondrial oxidative damage, ferroptosis, and lung inflammation in VILI

**Fig. 3:**
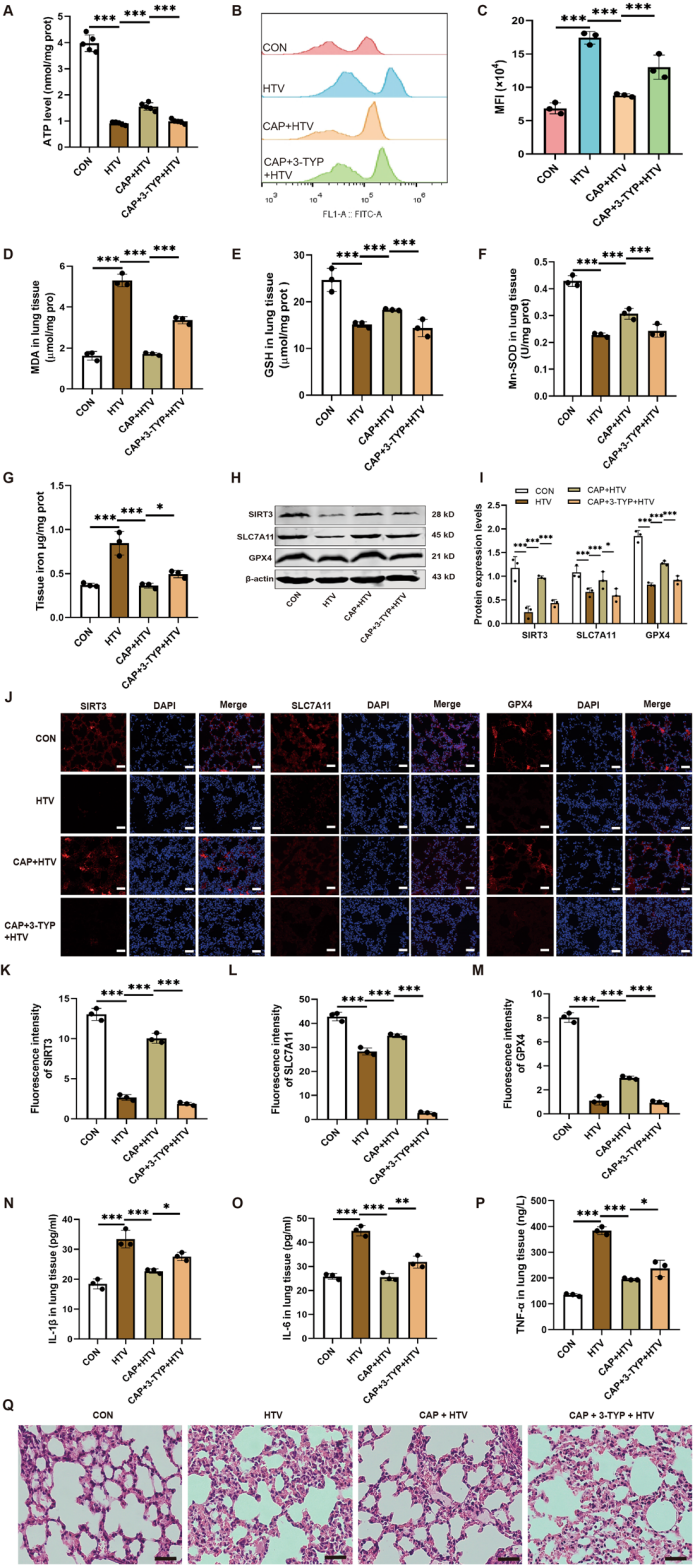
Pharmacological inhibition of SIRT3 alleviated the effect of capsaicin treatment on mitochondrial oxidative damage, ferroptosis, and lung inflammation in VILI. **A** Amount of ATP in the lung tissue (n = 5). **B**, **C** Flow cytometry analysis of reactive oxygen species (ROS) in monoplast suspension (n = 3). **D**–**F** Levels of MDA, GSH, and Mn-SOD in the lung tissue (n = 3). (G) Iron levels in the lung tissue (n = 3). **H** Representative western blots of SIRT3, SLC7A11, GPX4, and β-actin in the lung tissue. **I** Relative protein expression of SLC7A11 and GPX4 normalized to β-actin (n = 3). **J**–**M** Immunofluorescence staining and quantitative analysis of SIRT3, SLC7A11, and GPX4 in the lung tissue (n = 5, scale bar: 20 μm). **N** Levels of IL-1β in the lung tissue (n = 3). **O** Levels of IL-6 in the lung tissue (n = 3). **P** Levels of TNF-α in the lung tissue (n = 3). **Q** H&E staining in each group. Scale bar: 100 μm. Data are expressed as mean ± standard deviation. “*” indicates a significant difference between the corresponding groups (*p < 0.05, **p < 0.01, or ***p < 0.001)

The findings illustrated that, compared to the HTV group, the dysfunctional ATP synthesis, and high contents of ROS in lung tissue were reduced by CAP pretreatment under HTV conditions. However, these antagonistic effects of CAP were suppressed by the SIRT3 selective inhibitor 3-TYP treatment (Fig. 3A-C). Mitochondrial redox homeostasis was further confirmed by measurements of MDA (Fig. 3D), GSH (Fig. 3E), and Mn-SOD (Fig. 3F). The results showed that the addition of 3-TYP markedly blocked the protective effect of CAP against HTV-induced mitochondrial oxidative damage.

Additionally, iron levels in lung tissue (Fig. 3G) were reduced by CAP pretreatment under HTV conditions. However, after introducing 3-TYP pretreatment to the CAP group, total iron increased compared to the CAP-treated group. Furthermore, western blotting indicated that compared with CON mice, the expression degrees of SIRT3, SLC7A11, GPX4 proteins in the HTV group were dramatically reduced. CAP pretreatment could alleviate these results. However, 3-TYP preconditioning decreased SIRT3 protein activity and significantly reduced the beneficial influence of CAP on SLC7A11 and GPX4 protein in lung tissue (Fig. 3H, I). In line with the western blotting results, immunofluorescence staining and quantitative analysis showed that SIRT3, compared to the control group, SLC7A11 and GPX4 proteins were significantly reduced in the HTV group. While they were dramatically enhanced in the CAP + HTV team. Moreover, the expression levels of these proteins were dramatically decreased in the CAP + 3-TYP + HTV group compared to the CAP + HTV group (Fig. 3J–M). The results also showed that 3-TYP exacerbated lung injury, characterized by an increase in inflammatory cytokines (Fig. 3N–P) and lung tissue damage (Fig. 3Q).

In summary, these findings suggested that CAP alleviated VILI development by inhibiting ferroptosis in terms of maintaining mitochondrial function through SIRT3 activation.

### Overstretching of MLE12 cells activated cell injury and ferroptosis

**Fig. 4:**
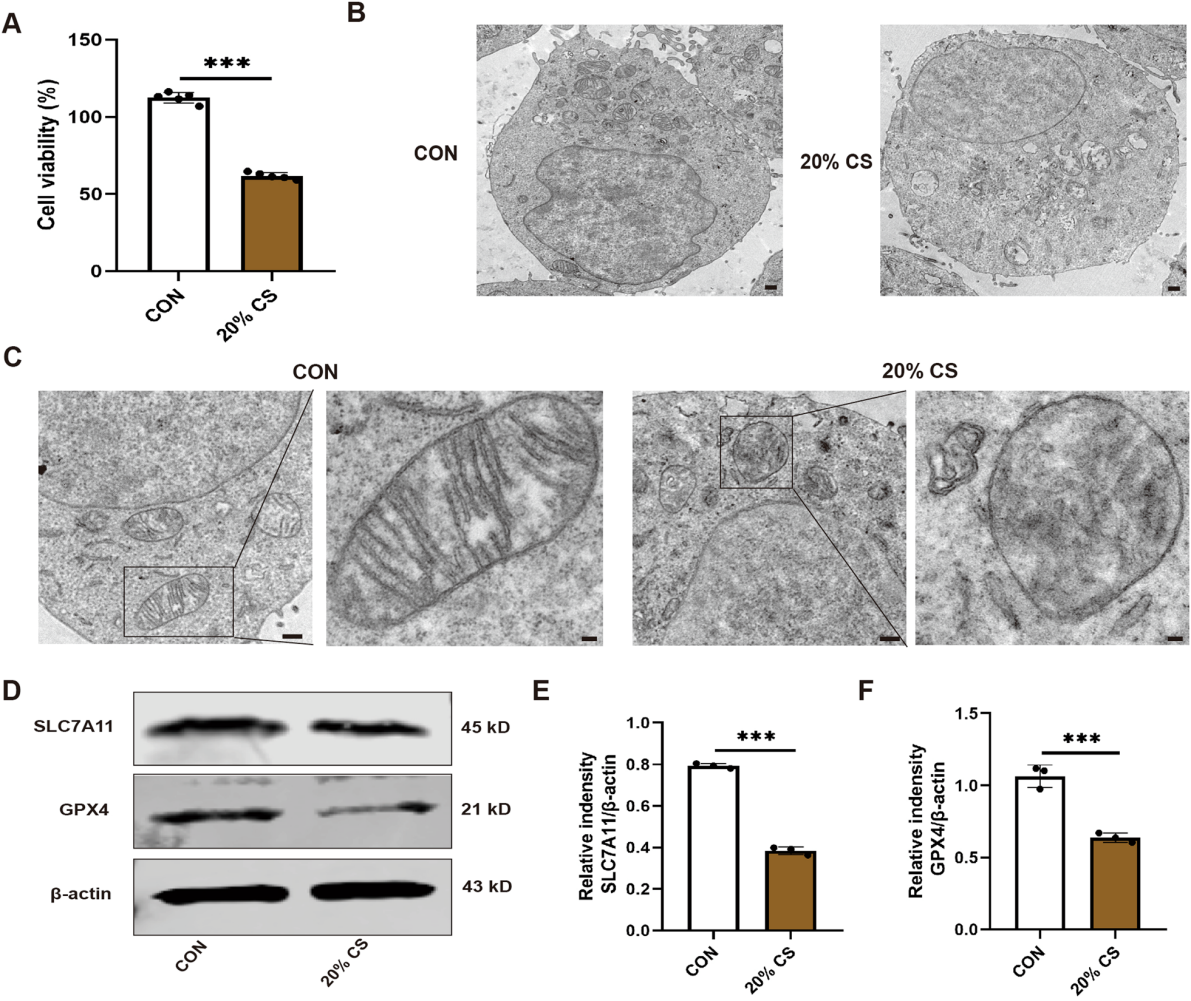
Overstretching of MLE12 cell activated cell injury and ferroptosis. **A** Cell viability of CS-MLE12 cells as observed using CCK8 assays (n = 5). **B** Representative transmission electron microscopy (TEM) images of MLE12 cells untreated or treated with 20% CS. Magnification: 1200 × , acceleration voltage: 80 kV. Scale bar: 5.0 μm. **C** Representative TEM images of the lung tissue sections. Magnification: 1200 × and acceleration voltage: 80 kV. Scale bar: 5.0 μm. Amplified pictures of mitochondria are labeled using black boxes, and the indicated area is shown at 6000 × magnification. Scale bar: 1.0 μm. **D** Representative western blots of SLC7A11, GPX4, and β-actin in MLE12 cells. **E**, **F** Relative protein expression of SLC7A11 and GPX4 was normalized to β-actin (n = 3). Data are expressed as mean ± standard deviation. “*” indicates a significant difference between the corresponding groups (*p < 0.05, **p < 0.01, or ***p < 0.001)

To further analyze the relationship between HTV-induced VILI and ferroptosis, in vitro experiments were conducted on MLE12 cells. A significant reduction in cell viability was observed in cells exposed to 20% CS for 4 h compared with nonstretched cells (Fig. 4A). TEM results indicated that cells in the 20% CS group showed obvious injury characterized by incomplete cell membranes and organelles, while those in the control group displayed intact cell morphology (Fig. 4B). We then investigated whether ferroptosis played a role in VILI in vitro. TEM results showed that 20% of the CS group exhibited typical mitochondria in the ferroptosis cells compared to the unstretched group (Fig. 4C). In addition, compared to the control team, the expression of SLC7A11 and GPX4 proteins in MLE12 cells was dramatically decreased after four hours of 20% CS exposure (Fig. 4D-F). The consequences illustrate that ferroptosis was induced and promoted cell injury when MLE12 cells were exposed to 20% CS for four hours.

### Capsaicin reduced MLE12 cell injury and ferroptosis after cyclic stretching

**Fig. 5:**
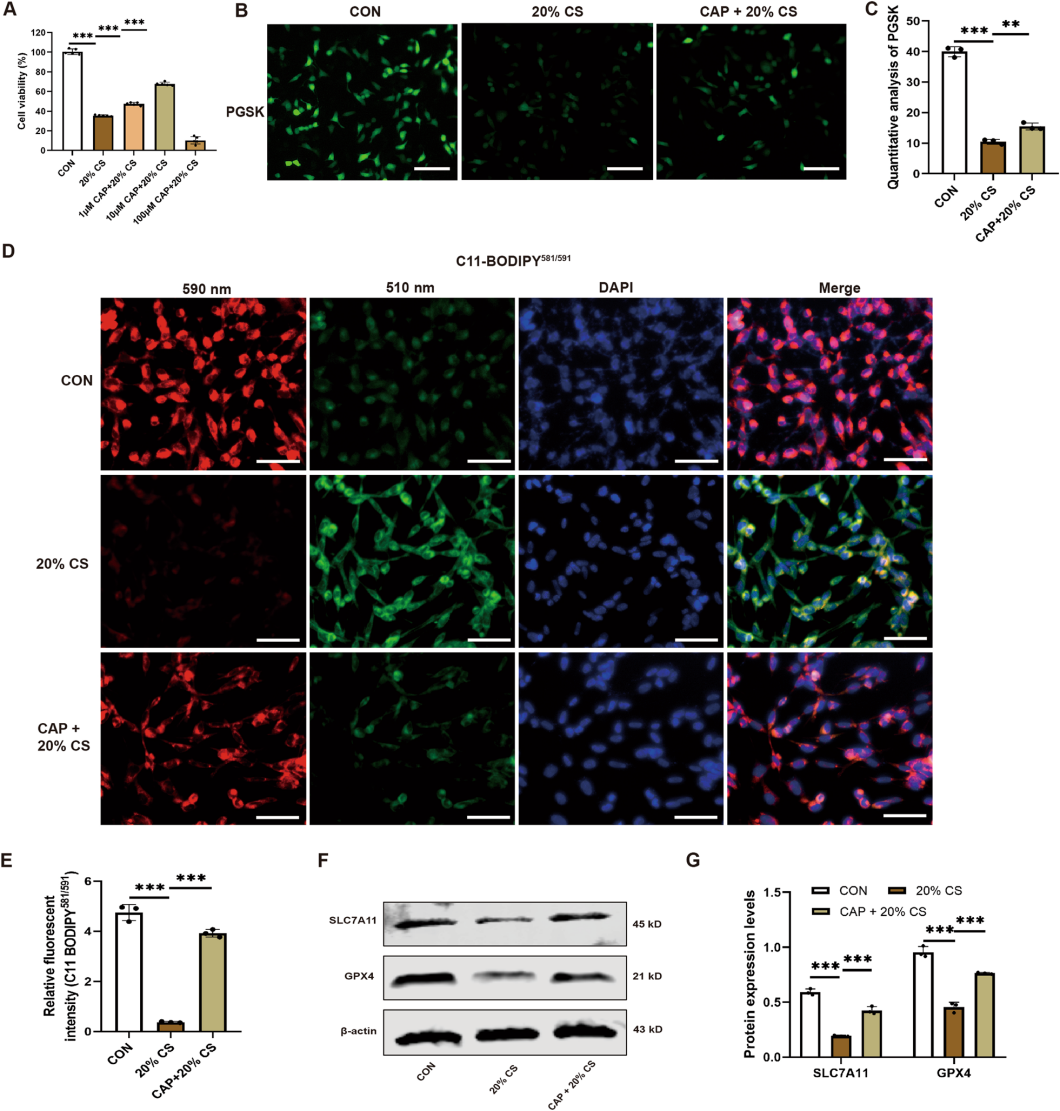
Capsaicin reduced MLE12 cell injury and ferroptosis after cyclic stretching. **A** Cell viability of CS-MLE12 cells after treatment with different doses of CAP (n = 5). **B**, **C** Immunofluorescence photomicrographs and quantitative analysis of PGSK (n = 3, Scale bar: 100 μm). **D**, **E** Immunofluorescence photomicrographs and quantitative analysis of C11-BODIPY.^581/591^ in MLE12 cells (n = 3, Scale bar: 50 μm). **F** Representative western blots of SLC7A11, GPX4, and β-actin in MLE12 cells. **G** Relative protein expression of SLC7A11 and GPX4 was normalized to β-actin (n = 3). Data are expressed as mean ± standard deviation. “*” indicates a significant difference between the corresponding groups (*p < 0.05, **p < 0.01, or ***p < 0.001)

To determine the optimal dose of CAP, a CCK8 assay was utilized to evaluate MLE12 cell viability. The most effective dose (10 μM) was identified in preliminary experiments using multiple concentrations of CAP (1, 10, and 100 μM; Fig. 5A). Cells in the 20% CS group displayed enhanced intracellular chelating iron (evaluated by PGSK assays) and lipid peroxidation (evaluated by C11-BODIPY^581/591^) compared with the nonstretched group. Surprisingly, CAP inhibited the levels of ferroptosis in MLE12 cells after four hours of 20% CS exposure, evident from the notable increase in fluorescence measurements of PGSK and C11-BODIPY^581/591^ (Fig. 5B–E). Consistent with the results, western blotting revealed that SLC7A11 and GPX4 proteins were significantly reduced in the 20% CS group compared with the control group. However, their levels were significantly increased in the CAP + 20% CS group (Fig. 5F, G). These data suggest that CAP enhanced MLE12 cell viability and diminished ferroptosis following CS.

### *Capsaicin further alleviated the mitochondrial oxidative damage *in vitro

**Fig. 6:**
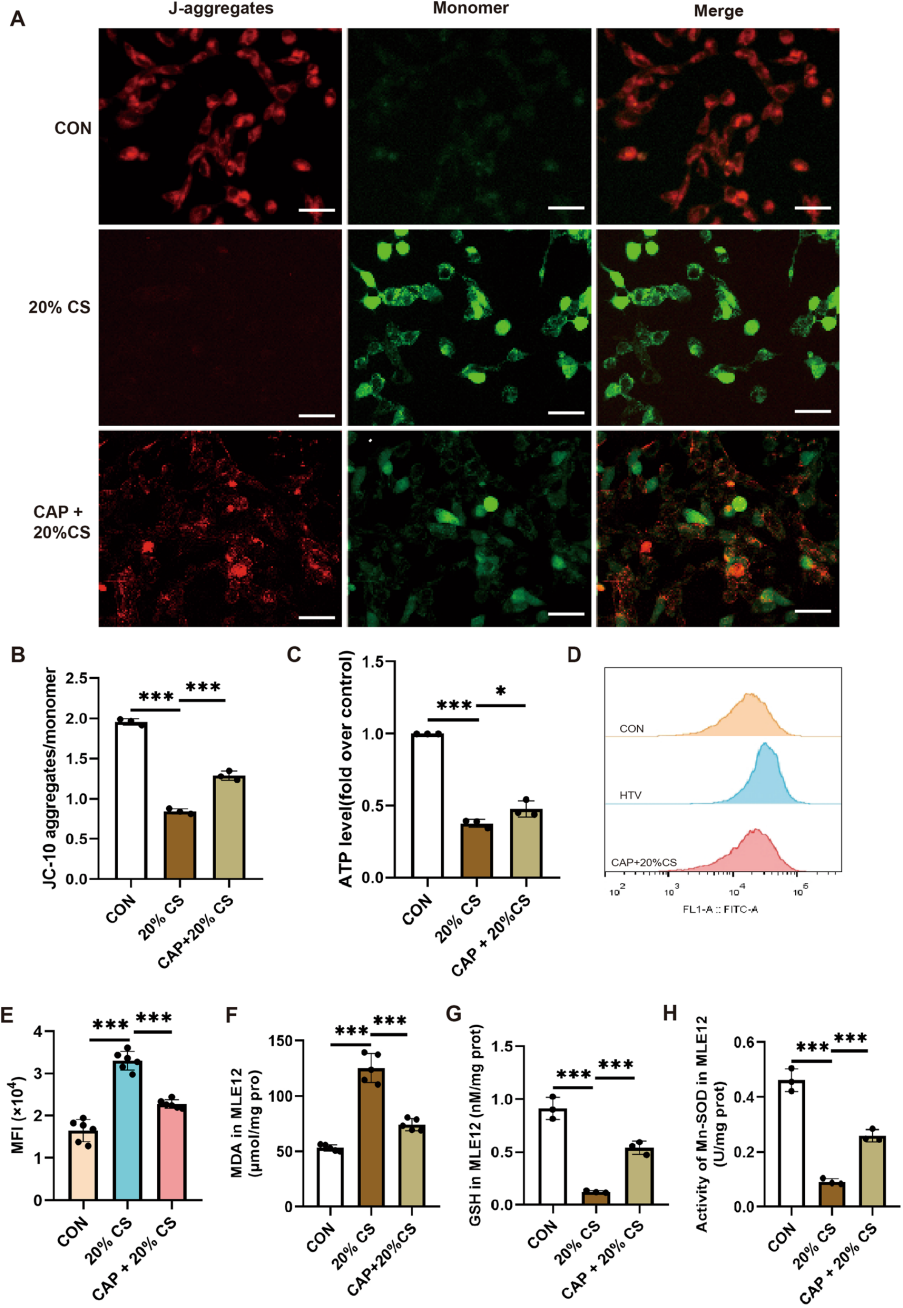
Capsaicin further alleviated the mitochondrial oxidative damage in vitro. **A** Levels of mitochondrial membrane potential (MMP) were noted by the decreased ratio of green (JC-10 monomer for low MMP)/red (J-aggregates for high MMP) fluorescence in MLE12 cells. Scale bar: 50 μm. **B** Quantitative analysis of MMP, as shown by JC-10 staining (n = 3). **C** Levels of ATP (n = 3). **D**, **E** Flow cytometry analysis of reactive oxygen species (ROS) in MLE12 cells (n = 5). (F) Levels of malondialdehyde (MDA) (n = 5). **G** Levels of glutathione (GSH) (n = 3). **H** Levels of Mn-SOD (n = 3). Data are expressed as mean ± standard deviation. “*” indicates a significant difference between the corresponding groups (*p < 0.05, **p < 0.01, or ***p < 0.001)

To investigate the effect of CAP on mitochondrial oxidative damage during overstretching of MLE12 cells, we measured changes in ATP, ROS, MDA, GSH, and Mn-SOD levels. Like the in vivo observations, a significant reduction in ATP and MMP and elevated ROS degrees were identified in the 20% CS group compared to those in the CON group (Fig. 6A–E). MDA degrees were higher in the 20% CS-treated cells compared to the CON cells (Fig. 6F), while GSH and Mn-SOD levels significantly declined (Fig. 6G, H). Notably, CAP pretreatment mitigated these effects, suggesting that CAP reduced HTV-induced mitochondrial oxidative damage (Fig. 6). Overall, our findings emphasized that ferroptosis occurred and promoted cell injury while we exposed MLE12 cells to 20% CS for four hours. Taken together, our findings demonstrated that CAP further diminished mitochondrial oxidative injury in the overstretching of MLE12 cells.

### *Knockdown SIRT3 attenuates the beneficial effect of capsaicin on mitochondrial oxidative damage and ferroptosis *in vitro

**Fig. 7:**
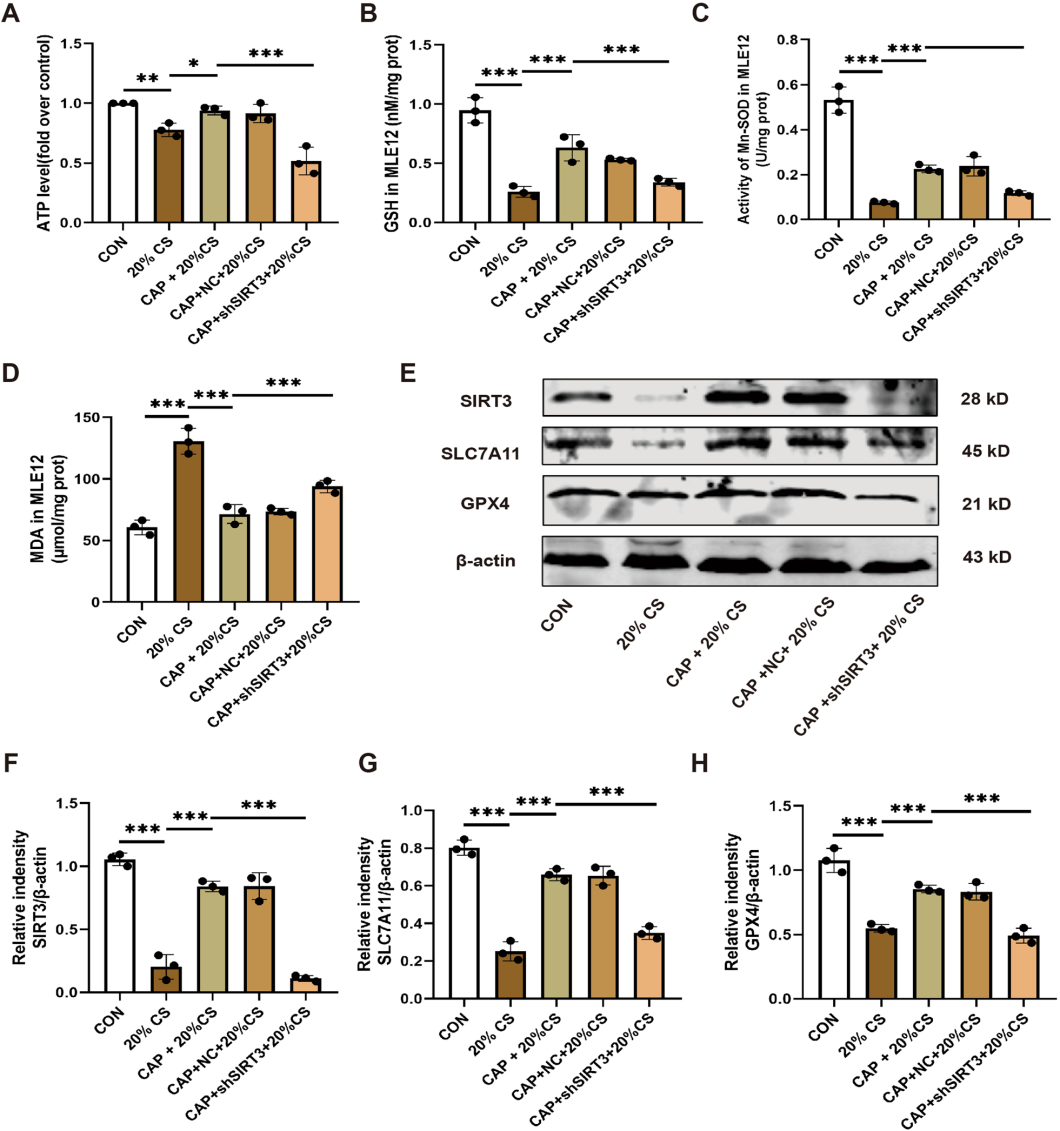
Knockdown SIRT3 attenuates the beneficial effect of capsaicin on mitochondrial oxidative damage and ferroptosis in vitro. **A** Levels of ATP (n = 3). **B** Levels of glutathione (GSH) (n = 3). **C** Levels of Mn-SOD (n = 3). **D** Levels of MDA (n = 3). **E** Representative Western blots of SIRT3, SLC7A11, GPX4, and β-actin in MLE12 cells. **F**–**H** Relative protein expression of SIRT3, SLC7A11, and GPX4 normalized to β-actin (n = 3). Data are expressed as mean ± standard deviation. “*” indicates a significant difference between the corresponding groups (*p < 0.05, **p < 0.01, or ***p < 0.001)

To identify the primary function of SIRT3 in adjusting mitochondrial redox homeostasis and inhibiting ferroptosis, we stably knocked down the SIRT3 gene in MLE12 cells using a lentiviral infection system (Supplementary Fig. 2A-C). Like the in vivo findings, in vitro results, including ATP (Fig. 7A), GSH (Fig. 7B), Mn-SOD (Fig. 7C), and MDA levels (Fig. 7D), displayed that the knockdown of SIRT3 dramatically impeded the protective effect of CAP against 20% CS-induced mitochondrial dysfunction and oxidative damage. The results also demonstrated that SIRT3 knockdown exacerbated ferroptosis in cells exposed to 20% CS, as characterized by reducing SLC7A11 and GPX4 protein levels (Fig. 7E–H).

## Discussion

Nowadays, MV remains an essential rescue therapy in critical care medicine; however, the improper application of MV can exacerbate VILI (Slutsky and Ranieri 2013). Investigating the pathogenesis and treatment of VILI is imperative. In this study, ventilation with a tidal volume of 20 ml/kg for 4 h and CS at 20% for 4 h were employed to establish in vivo and in vitro VILI models. It was done to analyze the impact of CAP on VILI and its underlying mechanism. Several key observations were made as follows: (1) Ferroptosis was observed, and it was found to enhance the inflammatory damage caused by MV with HTV in vivo or by overstretching MLE12 cells in vitro. (2) CAP offered protection against lung injury and inflammation during VILI. (3) CAP pretreatment safeguarded against lung injury and inflammation by inhibiting HTV or overstretching-induced ferroptosis. (4) CAP further reduced mitochondrial oxidative damage in VILI. (5) The inhibition of SIRT3 significantly blocked the protective effect of CAP on HTV or 20% CS-induced mitochondrial dysfunction and oxidative damage. (6) CAP alleviated VILI by suppressing ferroptosis, maintaining mitochondrial redox homeostasis through SIRT3-dependent mechanisms.

VILI has been extensively researched, and various harmful mechanisms have been identified. The occurrence of volutrauma, barotrauma, atelectrauma, oxygen toxicity, or sepsis has been postulated by scholars to culminate in a secondary injury termed biotrauma. This injury is typified by the release of mediators that stimulate the immune response (Xue et al. 2023). Ferroptosis is a form of programmed cell death induced by lipid peroxidation and dependent on iron, and it triggers immune responses (Ling et al. 2023; Zhou et al. 2020; Li et al. 2020a; Wu et al. 2021). The characteristics of it is to continuous release of damage-associated molecular patterns (DAMPs) and inflammatory cytokines (Ling et al. 2023). In our study, we discerned that HTV ventilation increased the levels of total iron, while the mRNA and protein levels of SLC7A11 and GPX4 were decreased in lung tissues. Both SLC7A11 and GPX4 are pivotal molecules linked with ferroptosis and can impede its occurrence. This aligns with our preceding research (Ling et al. 2023). In summary, our findings indicate that ferroptosis is involved in VILI, but the precise mechanism is unclear.

Mitochondria are pivotal organelles within cells, primarily responsible for generating energy that the cell requires, stored and transmitted in the form of ATP (Michaud et al. 2017; Vringer and Tait 2023). They play roles in diverse cellular metabolic processes, including lipid synthesis, intracellular material transport, maintaining cellular calcium balance, reducing oxidative stress, and promoting autophagy (Abate et al. 2020; Senft and Ronai 2015; Rodrigues and Ferraz 2020). In the context of HTV ventilation, it’s significantly concerning that it could cause mitochondrial damage, thereby disrupting their typical function. Such damage might manifest as compromised ATP synthesis, accumulation of ROS, and the loss of MMP, especially in alveolar type II cells (AT-IIs) (Koppula et al. 2018; Han et al. 2023; Liang et al. 2020). This has profound implications for lung injuries induced by MV. Cellular metabolism and ferroptosis share intricate interactions, with lipid ROS, primarily generated through oxygen/iron-driven metabolic processes, being essential for initiating ferroptosis(Chen et al. 2023). It is understood that ferroptosis is triggered by the cumulation of lipid peroxidation and it’s related to mitochondrial oxidative injury (Gao et al. 2019). This paper displayed that the HTV group showed smaller intracellular mitochondria, the density of mitochondrial ridge and mitochondrial membrane increased. Concurrently, the MDA content was elevated in the HTV-treated mice compared to the CON group, and these mice exhibited an oxidative state. Furthermore, compared with CON group, the extents of GSH and Mn-SOD in HTV group were dramatically reduced. These findings strongly suggest that mitochondrial redox imbalance induced by HTV or 20% CS may play a major role in promoting the onset or evolution of ferroptosis during VILI.

Consequently, our objective was to identify potent drugs capable of effectively mitigating VILI by inhibiting ferroptosis. CAP, or trans-8-methyl-N-vanillyl-6-nonenamide, is extracted from the fruits of chili pepper plants within the genus *Capsicum* (Sharma et al. 2013). This compound has garnered extensive research attention owing to its significant pharmacological properties. It has beneficial effects in a variety of conditions, ranging from obesity, cardiovascular and gastrointestinal disorders, diverse cancer types, and neurogenic bladder, to dermatological conditions(Chapa-Oliver and Mejia-Teniente 2016; Li et al. 2020b; Wang et al. 2022; Jaromi et al. 2018). Our results showed that intraperitoneal CAP administration for 3 consecutive days before MV decreased serum levels of pro-inflammatory cytokines involving IL-1β, IL-6, and TNF-α. It also resulted in reduced W/D ratios, cell counts in BALF, and lung injury scores. HE staining further highlighted that CAP mitigates lung damage induced by HTV. CAP has a significant role in ferroptosis. For instance, it has been discovered to promote ferroptosis in NSCLC by modulating SLC7A11/GPX4, and it can induce ferroptosis in glioma cells by the ACSL4/GPX4 pathway, thus providing antitumor benefits (Liu et al. 2022b; Hacioglu and Kar 2023). Concurrently, other research has observed that inhibiting ferroptosis can ameliorate intestinal ischemia–reperfusion injuries (Deng et al. 2021). The present study discovered that CAP could counteract the reduction in SLC7A11 and GPX4 levels caused by ferroptosis. Moreover, CAP was effective in reversing mitochondrial changes associated with ferroptosis, induced by HTV or 20% CS.

SIRT3, a soluble protein localized in the mitochondria, serves as a vital histone deacetylase (Li et al. 2021; Su et al. 2020). Its core function lies in regulating myriad cellular processes by deacetylating a plethora of protein substrates. By deacetylating these proteins, SIRT3 influences several crucial molecular processes, including metabolic homeostasis, oxidative stress, and cell death (Xu et al. 2021). Prior research has indicated that SIRT3 effectively curbs hyperoxia-induced ALI (Ning et al. 2022). As a mitochondrial protein, SIRT3 elevates the expression of Mn-SOD, attenuating the oxidative damage that arises from exposure to hyperoxia(Zhang et al. 2022b). The consequences mention that SIRT3 is a promising therapeutic target for hyperoxia-induced ALI treatment, given its potential antioxidative effects (Tian and Zhang 2018). It has been documented that activating the SIRT3/AMPK signaling pathway can lessen lung injury in ARDS, prevent apoptosis of endothelial cells, and fortify both mitochondrial and endothelial cell barrier integrity (Chen et al. 2023). Moreover, SIRT3’s ability to deacetylate and activate key antioxidant enzymes, such as superoxide dismutase (SOD2) and catalase, underscores its role in mitigating oxidative stress-a hallmark of ferroptosis (Liu, et al. 2022). Studies have demonstrated that SIRT3 influences ferroptosis through the PGC1α or SOD2 signaling pathways (Li et al. 2022; Jin, et al. 2023). Wang et. al, suggested that resveratrol ameliorated the generation of ROS through the Sirt3-FoxO3a pathway, thereby inhibiting the occurrence of ferroptosis (Wang, et al. 2023). Recent study confirmed that Sirt3 plays a crucial role in inhibiting oxidative stress-induced ferroptosis, thus improves Intervertebral disc degeneration and relives pain reaction (Zhu, et al. 2023). Interestingly, It has been suggested that Chronic dietary capsaicin could activate TRPV1 to restore Complex I function then up-regulating Sirt3 expression to reverse cardiac hypertrophy (Lang et al. 2015). Our results have shown that downregulated expression of SIRT3 dampened the beneficial effects of CAP treatment concerning mitochondrial oxidative damage, ferroptosis, and lung inflammation in VILI. But the intricate relationship between capsaicin and SIRT3 was not thoroughly investigated in this study, further studies are needed to further explore their relationship.

To summarize, our research illuminates the complex interrelation between VILI, ferroptosis, and the potential therapeutic roles of SIRT3 and CAP. Targeting ferroptosis could be a promising avenue for VILI treatment, and further research in this direction could yield valuable insights for managing this multifaceted ailment.

However, this study had some limitations. First, although mitochondrial oxidative damage is central to VILI, it is pivotal to recognize VILI's multifaceted and diverse pathogenesis, which encompasses other injury mechanisms that must not be disregarded. Second, the MLE12 cell line, representing prominent resident alveolar cells, is iron-ion-rich and crucial for iron metabolism. However, it remains unclear whether alveolar macrophages and other lung cells also undergo ferroptosis during VILI and what regulatory mechanisms are involved remain unclear. Lastly, subsequent research should explore the replicability of our findings in clinical studies on humans. In addition, research on the interaction between capsaicin and SIRT3 is currently limited, and further studies using gene knockdown mice may offer further insights and allow to dissect if other potential related effects have dominated VILI development and maintainment.

## Conclusions

CAP alleviated ferroptosis in VILI by improving SITR3 activity to suppress mitochondrial oxidative damage and maintain mitochondrial redox homeostasis.

## Supplementary Information

Supplementary material 1: **Figure 1. Ferroptosis was present during VILI. **Iron levels in the lung tissue of the CON, HTV, and HTV + Fer-1 groups.Relative mRNA levels of SLC7A11 and GPX4.Representative western blots of SLC7A11, GPX4, and β-actin in the lung tissue.Relative protein expression of SLC7A11 and GPX4 was normalized with β-actin.Representative transmission electron microscopyimages of lung sections derived from the CON, HTV, and HTV + Fer-1 groups. Magnification: 2000× and acceleration voltage: 80 kV. Scale bar: 5.0 μm. Amplified images of mitochondria are labeled using black boxes, and the indicated area is shown at 6000× magnification. Scale bar: 1.0 μm.Wet/dry ratios in the lung tissue.Infiltrating cell counts in BALF.H&E staining in each group. Scale bar: 100 μm.Pathological scores were assessed by H&E staining.Levels of IL-6 in the lung tissue.Levels of IL-1β in the lung tissue.Levels of TNF-α in the lung tissue. Data are expressed as mean ± standard deviation. “*” indicates a significant difference between the corresponding groups.

Supplementary material 2: **Figure 2. Gene knockdown of SIRT3 in MLE12 cells using a lentiviral infection system. **Relative mRNA levels of SIRT3.Representative Western blots of SIRT3 and β-actin in MLE12 cells.Relative protein expression of SIRT3 was presented to β-actin. Data are expressed as means ± SD. “*” indicates significant difference between corresponding group.

## Data Availability

The datasets used and/or analysed during the current study are available from the corresponding author on reasonable request.

## Abbreviations

VILI: Ventilator-induced lung injury
MV: Mechanical ventilation
CAP: Capsaicin
CS: Cyclic stretching
ARDS: Acute respiratory distress syndrome
ROS: Reactive oxygen species
GPX4: Glutathione peroxidases 4
SIRT3: Sirtuin 3
ALI: Acute lung injury
HTV: High tidal volume
TNF-α: Tumor necrosis factor-alpha
IL-6: Interleukin-6
IL-1β: Interleukin-1 beta
MMP: Mitochondrial membrane potential
MDA: Malondialdehyd
GSH: Glutathione
Mn-SOD: Manganese superoxide dismutase
Fer-1: Ferrostain-1
W/D: Wet/dry
TEM: Transmission electron microscopy
