# Supplementary figures and images for: Capsaicin mitigates ventilator-induced lung injury by suppressing ferroptosis and maintaining mitochondrial redox homeostasis through SIRT3-dependent mechanisms
Source: Mol Med. 2024 Sep 12;30:148. doi: 10.1186/s10020-024-00910-y (PMC11391744; doi:10.1186/s10020-024-00910-y)

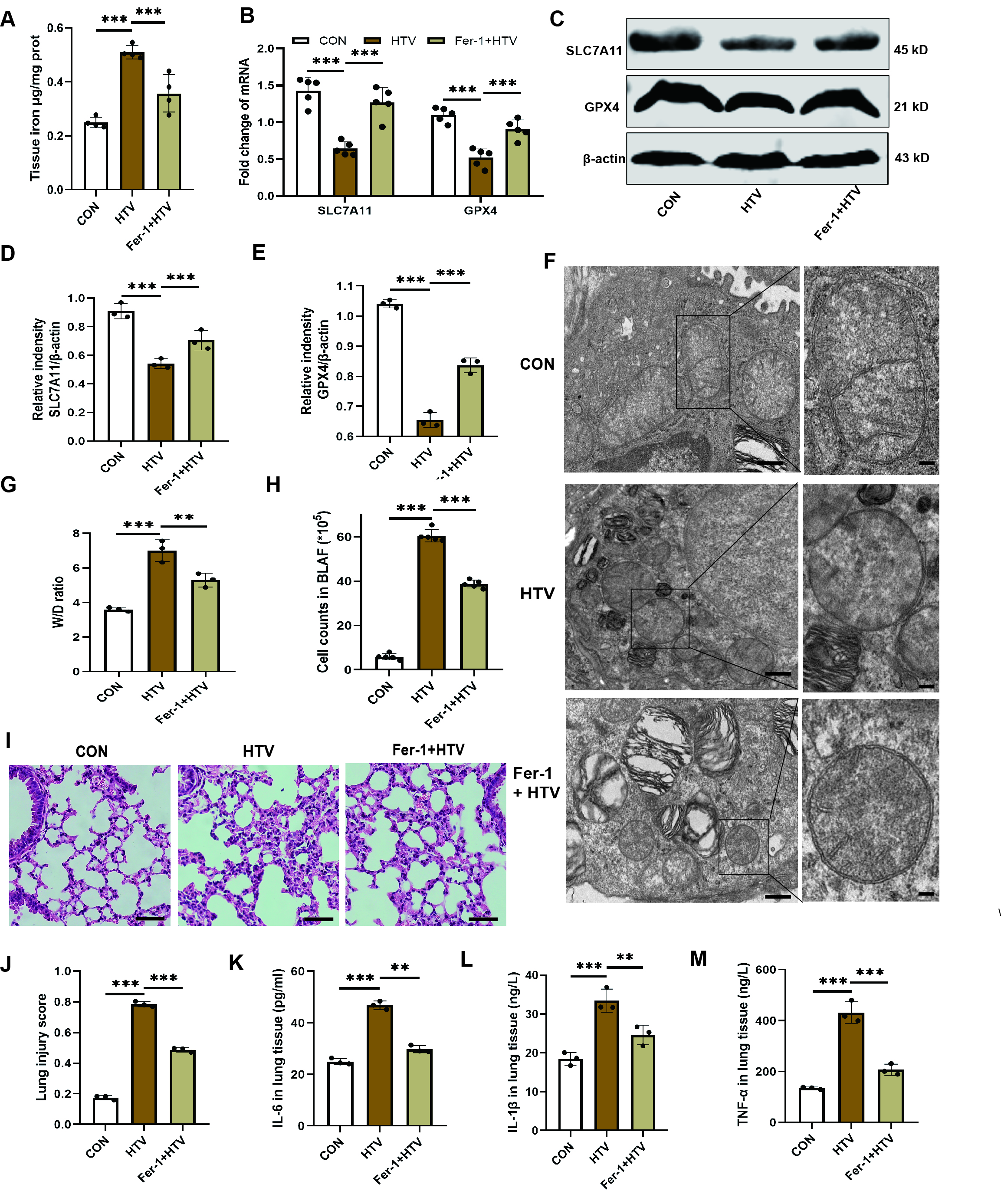

Supplement: Supplementary file 1 — Supplementary material 1: Figure 1. Ferroptosis was present during VILI. Iron levels in the lung tissue of the CON, HTV, and HTV + Fer-1 groups.Relative mRNA levels of SLC7A11 and GPX4.Representative western blots of SLC7A11, GPX4, and β-actin in the lung tissue.Relative protein expression of SLC7A11 and GPX4 was normalized with β-actin.Representative transmission electron microscopyimages of lung sections derived from the CON, HTV, and HTV + Fer-1 groups. Magnification: 2000× and acceleration voltage: 80 kV. Scale bar: 5.0 μm. Amplified images of mitochondria are labeled using black boxes, and the indicated area is shown at 6000× magnification. Scale bar: 1.0 μm.Wet/dry ratios in the lung tissue.Infiltrating cell counts in BALF.H&E staining in each group. Scale bar: 100 μm.Pathological scores were assessed by H&E staining.Levels of IL-6 in the lung tissue.Levels of IL-1β in the lung tissue.Levels of TNF-α in the lung tissue. Data are expressed as mean ± standard deviation. “*” indicates a significant difference between the corresponding groups. [file 10020_2024_910_MOESM1_ESM.jpg]

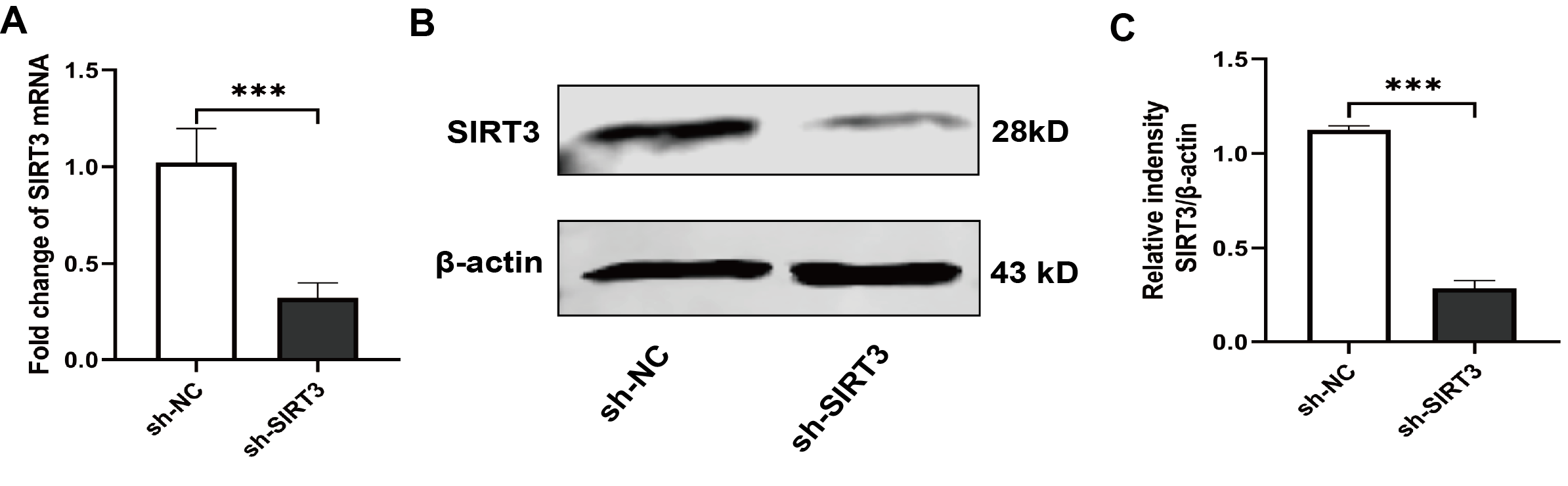

Supplement: Supplementary file 2 — Supplementary material 2: Figure 2. Gene knockdown of SIRT3 in MLE12 cells using a lentiviral infection system. Relative mRNA levels of SIRT3.Representative Western blots of SIRT3 and β-actin in MLE12 cells.Relative protein expression of SIRT3 was presented to β-actin. Data are expressed as means ± SD. “*” indicates significant difference between corresponding group. [file 10020_2024_910_MOESM2_ESM.tif]
